# Supplementary material for: Alum/CpG Adjuvanted Inactivated COVID-19 Vaccine with Protective Efficacy against SARS-CoV-2 and Variants
Source: Vaccines (Basel). 2022 Jul 29;10(8):1208. doi: 10.3390/vaccines10081208 (PMC9413105; doi:10.3390/vaccines10081208)
Supplement: Supplementary file 1 [file vaccines-10-01208-s001.zip › vaccines-1785742-supplementary.pdf]

Supporting Information

For

Alum/CpG adjuvanted inactivated COVID-19 vaccine with protective efficacy against SARS-CoV-2 and variants

Yuntao Zhang[1,2,†], Xiaotong Zheng[1,†], Wang Sheng[3], Hongyang Liang[1], Yuxiu Zhao[1], Xiujuan Zhu[1], Rong Yang[1], Yadan Zhang[1], Xiaofei Dong[1], Weidong Li[1], Fei Pei[1], Ling Ding[1], Zhen Chang[1], Li Deng[1], Guangying Yuan[1], Zhaona Yang[1], Di Zhu[1], Xiaoming Yang[1,2,*], Hui Wang[1,*]

[1]Beijing Institute of Biological Products Company Limited, Beijing, China

[2]China National Biotec Group Company Limited, Beijing, China

[3]Beijing University of Technology, Beijing, China

[†]These authors contributed equally

*Correspondence: wanghui_bsy@163.com (H.W.), yangxiaoming@sinopharm.com (X.Y.)

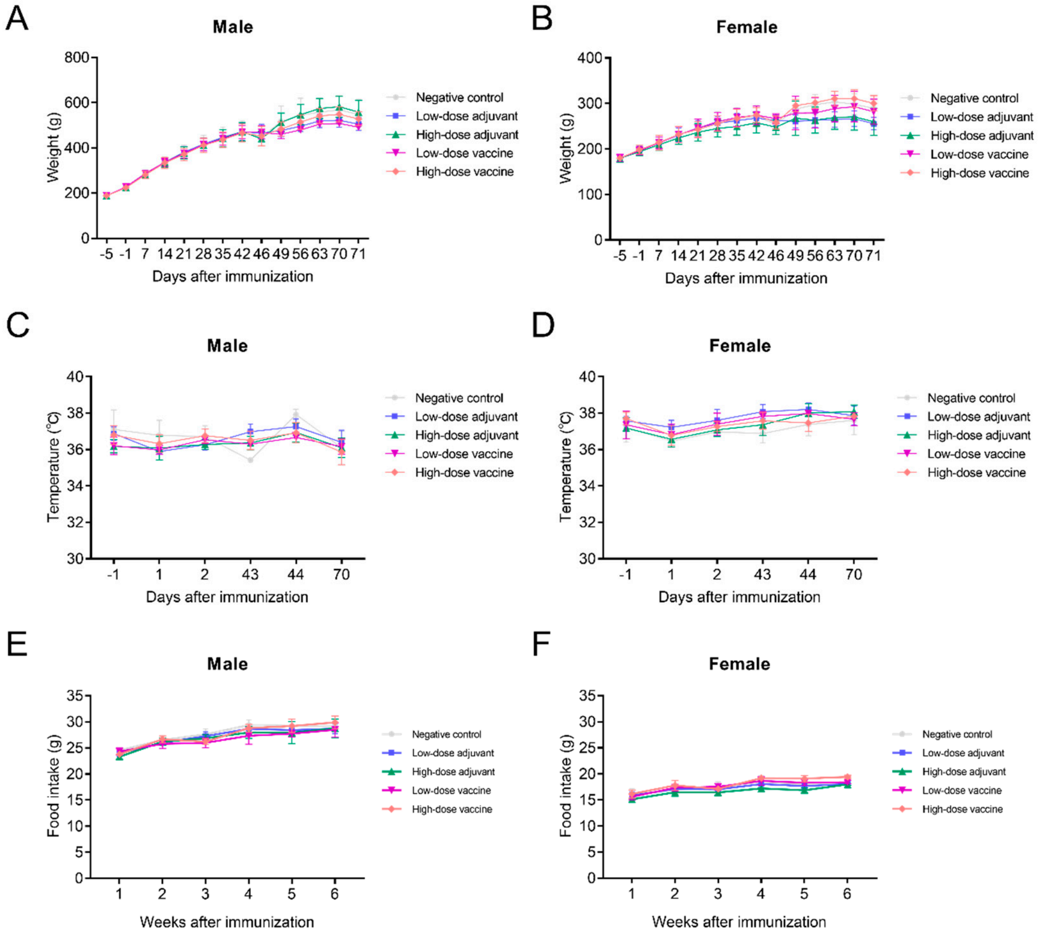

Supplementary Figure S1. Clinical evaluation of rats in different groups. A and B Body weight (n=5~15). C and D Body temperature (n=5). E and F Food intake (n=15).

Supplementary Table S1. Pathological observation of bone marrow and spleen at day 46. The lesions were graded according to a 5-point scale (slight, mild, moderate, marked, severe).

**Gender**	**Male**					**Female**				
**Groups**	**1**	**2**	**3**	**4**	**5**	**1**	**2**	**3**	**4**	**5**
**Number of animals**	**10**	**10**	**10**	**10**	**10**	**10**	**10**	**10**	**10**	**10**
**Bone marrow**										
Increased cellularity, myeloid	0	2	3	3	6	0	5	7	5	5
slight	0	2	3	3	4	0	5	5	5	4
mild	0	0	0	0	2	0	0	2	0	1
**Spleen**										
Hyperplasia, germinal center, white pulp	0	0	0	1	4	0	0	0	0	2

Gender	Male					Female				
**Groups**	**1**	**2**	**3**	**4**	**5**	**1**	**2**	**3**	**4**	**5**
**Number of animals**	**10**	**10**	**10**	**10**	**10**	**10**	**10**	**10**	**10**	**10**
slight	0	0	0	1	4	0	0	0	0	2

Supplementary Table S2. Pathological observation of inguinal lymph nodes at day 46. The lesions were graded according to a 5-point scale (slight, mild, moderate, marked, severe).

Gender	Male					Female				
**Groups**	**1**	**2**	**3**	**4**	**5**	**1**	**2**	**3**	**4**	**5**
**Number of animals**	**10**	**10**	**10**	**10**	**10**	**10**	**10**	**10**	**10**	**10**
**Lymph nodes, groin**										
Hyperplasia, germinal center, cortex	0	0	1	2	3	0	3	1	2	7
slight	0	0	1	2	0	0	3	1	2	5
mild	0	0	0	0	3	0	0	0	0	2
Plasmacytosis, medulla	0	1	0	2	4	0	0	1	1	6
slight	0	1	0	2	2	0	0	1	1	2
mild	0	0	0	0	1	0	0	0	0	4
moderate	0	0	0	0	1	0	0	0	0	0
Epithelioid cell aggregates	0	0	0	0	0	0	0	2	0	0
slight	0	0	0	0	0	0	0	1	0	0
mild	0	0	0	0	0	0	0	1	0	0

Supplementary Table S3. Pathological observation of administration site at day 46. The lesions were graded according to a 5-point scale (slight, mild, moderate, marked, severe)

Gender	Male					Female				
**Groups**	**1**	**2**	**3**	**4**	**5**	**1**	**2**	**3**	**4**	**5**
**Number of animals**	**10**	**10**	**10**	**10**	**10**	**10**	**10**	**10**	**10**	**10**
**Local administration**										
Granulomatous inflammation	0	9	10	10	9	0	10	10	10	10

Gender	Male					Female				
**Groups**	**1**	**2**	**3**	**4**	**5**	**1**	**2**	**3**	**4**	**5**
**Number of animals**	**10**	**10**	**10**	**10**	**10**	**10**	**10**	**10**	**10**	**10**
slight	0	2	1	3	0	0	0	1	4	0
mild	0	5	4	4	3	0	5	3	5	2
moderate	0	2	5	3	4	0	5	6	1	8
marked	0	0	0	0	2	0	0	0	0	0
Edema, interstitial	0	4	8	6	6	0	5	7	8	10
slight	0	2	5	2	0	0	3	3	3	0
mild	0	0	3	4	6	0	2	4	5	8
moderate	0	2	0	0	0	0	0	0	0	2
Necrosis, interstitial	0	3	4	1	1	0	2	1	1	1
slight	0	0	2	1	0	0	1	1	1	0
mild	0	3	2	0	1	0	1	0	0	1
Degeneration/necrosis, muscle fibers	0	4	5	3	5	2	3	3	2	7
slight	0	4	5	3	5	2	3	1	2	6
mild	0	0	0	0	0	0	0	2	0	1
Inflammatory cell infiltration, interstitial	10	5	8	9	6	8	3	9	9	7
slight	8	3	4	5	3	6	3	1	3	0
mild	2	2	4	4	3	2	0	8	6	7
Granulation tissue	0	0	0	1	2	0	3	2	3	0
slight	0	0	0	1	0	0	1	2	1	0
mild	0	0	0	0	1	0	2	0	2	0
moderate	0	0	0	0	1	0	0	0	0	0
